# Supplementary material for: CD300e modulates metabolic programs in adipose tissue macrophages during obesity
Source: Cell Death Dis. 2026 Jun 15;17(1):747. doi: 10.1038/s41419-026-08974-w (PMC13493925; doi:10.1038/s41419-026-08974-w)
Supplement: Supplementary file 1 [file 41419_2026_8974_MOESM1_ESM.docx]

**CD300e modulates metabolic programs in adipose tissue macrophages during obesity**

Sara Coletta^1*^, Simone Pizzini^1^, Stefania Vassallo^1^, Elisabetta Trevellin^2^, Annica Barizza^1^, Sofia Giacometti^1^, Gabriele Sales^1^, Mattia Laffranchi^3^, Silvano Sozzani^3^, Marta Murgia^4,5^, Agnese De Mario^4^, Cristina Mammucari^4^, Fabrizia Carli^6^, Camilla Bean^7,8^, Amalia Gastaldelli^6^, Roberto Vettor^2,7^, Marina de Bernard^1*^

^1^Department of Biology, University of Padova, Padova, Italy

^2^Department of Medicine (DIMED), University of Padova, Padova, Italy

^3^Department of Molecular Medicine, Sapienza University of Rome, Laboratory Affiliated to Istituto Pasteur-Fondazione Cenci Bolognetti, Rome, Italy^.^

^4^Department of Biomedical Sciences, University of Padova, Padova, Italy

^5^Department of Proteomics and Signal Transduction, Max‑Planck-Institute of Biochemistry, Martinsried, Germany

^6^Cardiometabolic Risk Unit, Institute of Clinical Physiology, National Research Council-CNR, Pisa, Italy

^7^Departmental Faculty of Medicine, UniCamillus-Saint Camillus International University of Health and Medical Sciences, Rome, Italy

^8^Veneto Institute of Molecular Medicine, Padova, Italy

^9^Center for Metabolic and Nutrition Related Diseases - Humanitas Research Hospital Rozzano, Milano, Italy

*Correspondence: Sara Coletta, Department of Biology, Via Ugo Bassi 58b, 35131 Padova, Italy. Phone: +39.049.8276308. Email: [sara.coletta@unipd.it](mailto:sara.coletta@unipd.it) & Marina de Bernard, Department of Biology, Via Ugo Bassi 58b, 35131 Padova, Italy. Phone: +39.049.8276309. Email: [marina.debernard@unipd.it](mailto:marina.debernard@unipd.it) (M.d.B.)

**Supplementary Files:**

- Supplementary Figure 1
- Supplementary Figure 2
- Supplementary Figure 3
- Supplementary Figure 4
- Supplementary Table S2
- Supplementary Table S3
- Supplementary Table S4

**Supplementary Figure 1. Effect of Cd300e deficiency on food intake, respiratory exchange ratio, energy expenditure, and thermogenesis.**

(A) Daily food intake for individual mice of the indicated genotypes. Each dot represents one mouse (n=4 per group); bars indicate mean ± SEM.

(B) Left panel: Respiratory exchange ratio (RER) over 6 days during light and dark cycles in WT and *Cd300e^-/-^* mice measured in metabolic chambers. Dark phases are indicated in grey; data are represented as mean ± SEM (n = 4 mice per genotype). Right panel: mean of daily RER for each mouse, bars indicate mean ± SEM (n = 4 mice per genotype).

(C) Left panel: Energy expenditure (EE) over 6 days during light and dark cycles in WT and *Cd300e^-/-^* mice measured in metabolic chambers. Dark phases are indicated in grey; data are represented as mean ± SEM (n = 4 mice per genotype). Right panel: mean of daily EE for each mouse, bars indicate mean ± SEM (n = 4 mice per genotype).

(D) mRNA expression of *Ucp1* was evaluated by qRT-PCR in BAT of WT and *Cd300e^-/-^* mice. Data were normalized on 18S as endogenous reference gene and are expressed as 2^-ΔΔCt^ relative to WT mice. Data are expressed as mean ± SEM (n=6 per genotype).

**Supplementary Figure 2. Blood immune cell composition is not influenced by the diet regimen and the absence of Cd300e.** The percentage of CD11b^+^ myeloid cells, Ly6G^-^Ly6C^+^ monocytes and Ly6G^+^Ly6C^+^ neutrophils, CD4^+^ and CD8^+^ T lymphocytes, and CD19^+^ B lymphocytes were determined within total blood CD45^+^ leukocytes collected from WT and *Cd300e^-/-^* mice after 16 weeks of CFD or HFD feeding. Data are expressed as percentage (%) of CD45^+^ cells (mean ± SEM of n=16/genotype of CFD-fed mice and n=14/genotype of HFD-fed mice).

**Supplementary Figure 3. Proteomic analysis of *Cd300e^-/-^* ATMs.**

(A) Schematic overview of the mass-spectrometry-based proteomic workflow. Created with BioRender.com.

(B) PCA analysis of the proteomic data.

(C) Volcano plot showing enriched proteins between genotypes at T_0_ (left panel) or under HFD (right panel). Dotted lines represent the applied significance thresholds (p value = 0.05).

**Supplementary Figure 4. Gating strategy for the identification of the circulating monocytes subsets.** Forward and side scatter were used to isolate the interesting population. Subsequently T, B and NK cells were excluded and the CD3^-^, CD19^-^ and CD56^-^ cells identified within the single cell and live cell gates. Monocytes were identified and divided into classical (CD14^+^, CD16^-^), intermediate (CD14^+^, CD16^+^) and non-classical monocytes (CD14^-^, CD16^+^). Then, any further contamination of NK cells, neutrophils (CD16^+^, HLA-DR^-^) and B cells (HLA-DR^+^, CD14^-^) has been excluded and within the three subclasses of monocytes the fluorescence intensity of CD300e has been analyzed.

|  | **Lean** | **Obese** |
| --- | --- | --- |
| **Sex** | **18 F and 17 M** | **24 F and 19 M** |
| **Age (mean ± SEM)** | **(34.6 ± 1.6)** | **(42.9 ± 1.4)** |
| **Height (mean ± SEM)** | **(172.8 ± 1.8)** | **(169.8 ±1.4)** |
| **Weight (mean ± SEM)** | **(64.8 ±2)** | **(122.5 ± 3.5)** |
| **BMI (mean ± SEM)** | **(21.6 ±0.3)** | **(42.5±1.1)** |

**Supplementary Table S2. Clinical characteristics of lean and obese patients.**

| **siRNA** | **Sequence** |
| --- | --- |
| *Hs_IREM2_1* | TCCCATCTTCCTGGTGGTGAA |
| *Hs_IREM2_2* | CAGAACCTCAATGAAGATGAT |
| *Hs_IREM2_3* | GAGCATGTACAAGGGATATAA |
| *Hs_IREM2_5* | CAGTGTGGTGTCAGTATGAGA |

**Supplementary Table S3. SiRNA sequence used for CD300e silencing.**

| **Primer** | **Sequence 5’ – 3’** |
| --- | --- |
| h β-actin FW | TGAGATGCGTTGTTACAGGA |
| h β-actin RV | ACGAAAGCAATGCTATCA |
| h *CD300e* FW | GGGAGGTGTTGACCCAAAAT |
| h *CD300e* RV | AGGACCACGAGCAGGAAGT |
| m β-actin FW | GATTACTGCTCTGGCTCCTAGC |
| m β-actin RV | GACTCATCGTACTCCTGCTTGC |
| m 18S FW | TGTCTCAAAGATTAAGCCATGC |
| m 18S RV | GCGACCAAAGGAACCATAAC |
| m *Cd300e* FW | CCAGTGAGGCAGGAGGATGAG |
| m *Cd300e* RV | AGAGACAAACAACCTTGGAAGCAGAG |
| m *Il6* FW | TAATTCATATCTTCAACCAA |
| m *Il6* RV | TCCTTAGCCACTCCTTCTGT |
| m *Tnf* FW | ACTGAACTTCGGGGTGAT |
| m *Tnf* RV | CTGAGTGTGAGGGTCTGG |
| m *Il1b* FW | CTGCTTCCAAACCTTTGACC |
| m *Il1b* RV | AGCTTCTCCACAGCCACAAT |
| m *Acc* FW | GGCCAGTGCTATGCTGAGAT |
| m *Acc* RV | AGGGTCAAGTGCTGCTCCA |
| m *Atgl* FW | CAGCACATTTATCCCGGTGTAC |
| m *Atgl* RV | AAATGCC GCCATCCACATAG |
| m *Srebp1* FW | GCGCTACCGGTCTTCTATCA |
| m *Srebp1* RV | TGCTGCCAAAAGACAAGGG |
| m *Chrebp* FW | AAGTCCTTGGTCGGGAAGTATACA |
| m *Chrebpv* RV | ACTCCCTCAAAGTCATCACAAACA |
| m *Fas* FW | CTGCGGAAACTTCAGGAAATG |
| m *Fas* RV | GGTTCGGAATGCTATCCAGG |

**Supplementary Table S4. Primers’ sequence used for qRT-PCR.**
